# Analyzing the influence of environment, demographic and socio-economic factors on *Aedes albopictus* (Diptera: Culicidae) mosquito density at the micro-level using XGBoost and SHAP

**DOI:** 10.1186/s13071-025-07220-0

**Published:** 2026-01-09

**Authors:** Junyi Yao, Zijun Zhou, Hongxia Liu, Shenjun Yao, Jianping Wu

**Affiliations:** 1 Shanghai Municipal Center for Disease Control and Prevention, Shanghai, China; 2 Key Laboratory of Geographic Information Science, Ministry of Education, Shanghai, China; 3 School of Geographic Sciences, East China Normal University, Shanghai, China; 4 Key Laboratory of Spatial-temporal Big Data Analysis and Application of Natural Resources in Megacities, Ministry of Natural Resources, Shanghai, China; 5 Institute of Cartography, East China Normal University, Shanghai, China

**Keywords:** *Aedes albopictus*, Mosquito density prediction, XGBoost, SHAP, Interpretability analysis, Public health, Machine learning

## Abstract

**Background:**

Effective mosquito control in urban areas requires understanding of how climatic, ecological and socioeconomic factors shape vector abundance. However, most studies use linear or opaque models that overlook nonlinear relationships between environmental conditions and *Aedes albopictus* density. These complex associations remain insufficiently characterized in highly urbanized settings, where interacting environmental and human factors jointly influence mosquito habitats.

**Methods:**

We trained a random forest model, an XGBoost model with a default squared-error objective and an XGBoost model with a Poisson count objective using adult *Aedes albopictus* monitoring data collected across Shanghai from April to November 2023. Model performance was evaluated with RMSE, MAE, R^2^ and Poisson deviance, and temporally blocked cross-validation was applied to assess temporal generalizability. SHAP analysis was used to interpret variable importance and contribution patterns. To examine operational relevance, we additionally evaluated hotspot localization accuracy using July 2024 data.

**Results:**

On the independent test set, the XGBoost-Poisson model achieved the best overall accuracy (*R*^2^ = 0.73, Poisson deviance = 4.52). SHAP analysis identified the 14-day temperature lag as the dominant predictor, followed by a slight negative population density and compulsory completion of education. Precipitation and NDVI showed smaller positive contributions. Age structure variables exhibited nonlinear trends—with an inverted-U shape for children, a declining pattern for older adults and a shallow U shape for building height. By site type, mosquito density tended to be higher near schools, livestock sheds and office areas and lower in residential, farmhouse, park and hospital environments. Under temporally blocked cross-validation, the model retained moderate temporal generalization. In out-of-time hotspot validation, the top 10% of sites captured 41–50% of hotspots, rising to 60–68% at 25% coverage, suggesting moderate spatial localization.

**Conclusions:**

The framework identified key environmental and socioeconomic drivers of *Aedes albopictus* density in Shanghai. Despite moderate temporal generalization, it provides interpretable, fine-scale insights to guide targeted vector control and inform urban mosquito management in dense metropolitan settings. Future research should validate the framework across additional seasons and diverse urban contexts, incorporate finer environmental and infrastructural data and enhance uncertainty quantification for improved interpretive robustness.

**Graphical abstract:**

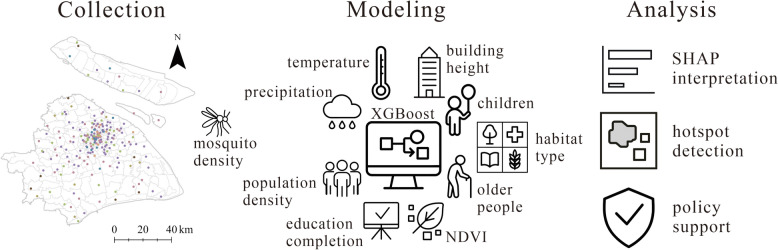

## Background

Mosquitoes disrupt daily life through blood-feeding and transmit more than 40 human diseases [1], including malaria, yellow fever, dengue, West Nile virus and Japanese encephalitis [2]. Among these mosquitoes, *Aedes albopictus* plays a significant role in disease transmission [3], contributing to the spread of dengue fever across more than 120 countries, spanning the Western Pacific, Southeast Asia, the Americas, Europe and Africa [4, 5]. In China, *Ae. albopictus* is widely distributed across tropical and temperate regions [6], making it the primary vector for dengue [7], Zika virus [8] and chikungunya [9, 10]. Effective vaccines are available for yellow fever [11] and Japanese encephalitis [12]; dengue vaccines exist but have age- and setting-specific indications and programmatic constraints [13]. Accordingly, vector control remains central to prevention in many urban settings [14].

Understanding *Ae. albopictus* density (e.g. the number of adult *Ae. albopictus* mosquitoes observed per trap) dynamics is crucial for vector surveillance and control [15]. Previous studies have found that meteorological factors strongly influence mosquito density. Temperature affects mosquito development rates, with higher temperatures accelerating larval development and adult emergence [16–23]. Precipitation is another major determinant, as it creates water-filled habitats conducive to mosquito breeding [17, 24–29]. Relative humidity (RH) usually facilitates adult mosquito survival [30]; however, a Shanghai study found no significant association, probably because consistently high RH values reduce variability and detectable associations [31–33]. These relationships are often nonlinear. For instance, temperature effects commonly peak at approximately 26–30 °C [18, 20, 22, 23], and weather-abundance associations often exhibit short lags (≈1–4 weeks) in adult abundance [16, 34, 35]. At urban scales, some studies and reviews have reported stronger associations for environmental and socioeconomic variables, such as population density and social deprivation, than for meteorological factors [31, 36, 37], although findings vary by context. For instance, environmental variables such as NDVI and population density usually positively influence mosquito density. While the former provides resting and breeding sites [38–40], the latter increases host availability and encounter rates [41].

While core environmental and meteorological factors such as NDVI, population density and temperature have been widely studied, the roles of other urban environmental variables—such as education level, population structure, building height and mosquito habitat type—remain insufficiently explored. We hypothesize that several urban covariates relate to *Ae. albopictus* density through distinct pathways. Education level (compulsory completion of education) may proxy broader socioeconomic and built-environment conditions and container-management practices, such as water storage and waste handling, which shape source availability. Population structure, reflected in the proportions of children and older adults, may influence human-mosquito contact opportunities through differences in outdoor activity and protection practices. Building height may modify microclimate and vertical habitat by providing shade, wind sheltering and heat retention on balconies or rooftops, thereby affecting resting and breeding suitability. Mosquito habitat types may reflect differences in source abundance and maintenance regimes across settings such as schools, construction sites and livestock sheds, which may influence containers and shape local breeding conditions. These considerations guide our choice of covariates and the interpretation of model outputs in this study.

This study focuses on the trap index of adult *Ae. albopictus*, which is denoted as the number of *Ae. albopictus* captured per CO_2_-baited trap during the 6-h sampling period, and explores its potential influential factors in Shanghai, China. Located on the eastern coast of China, Shanghai has a typical subtropical monsoon climate characterized by hot summers and heavy rainfall, which together create favorable conditions for mosquito breeding. As one of China’s most populous and highly urbanized cities, it provides a distinctive setting for mosquito research, featuring dense populations, diverse land-use patterns and complex urban infrastructure. The city’s modern environment and high human mobility further complicate vector control efforts. In 2017, Shanghai reported its first locally transmitted dengue fever case, underscoring the urgent need to strengthen mosquito-borne disease prevention and control strategies [42]. These climatic, demographic and environmental complexities make Shanghai an ideal case for investigating the drivers of mosquito density and developing optimized control measures applicable to other megacities facing similar challenges.

While traditional statistical models, such as regression analysis [16, 43, 44], time series modeling [45, 46] and ecological niche models [17, 38, 47], have been widely used and remain valuable in certain contexts, they often face limitations when addressing nonlinear relationships and high-dimensional datasets typical of urban mosquito density prediction. Recent studies have increasingly adopted machine learning techniques—such as k-nearest neighbors (k-NN), artificial neural networks (ANN), support vector machines (SVM) and random forests (RF)—to model complex interactions [48–51], consistently improving predictive accuracy and robustness to environmental variability [49, 52] and better capturing nonlinear associations often underrepresented in conventional frameworks [16].

Although ensemble learning methods such as random forests (RF) can effectively capture complex and high-order interactions, they may be limited by bias-variance trade-offs and the lack of explicit regularization, which can lead to less smooth or overly variable functional fits. To address these issues, we employ Extreme Gradient Boosting (XGBoost), which incorporates regularized boosting and early-stopping mechanisms to enhance both predictive stability and computational efficiency [53]. To mitigate the “black box” concern, we integrate SHapley Additive exPlanations (SHAP) for post hoc interpretability, which is effective for explaining vector modeling results [54–56]. Although XGBoost has rarely been applied to mosquito density per se, its strong performance in malaria risk modeling supports its suitability for our setting [57, 58].

The primary aim of this study is to identify and interpret the environmental and socio-demographic factors associated with fine-grained spatial variation in *Ae. albopictus* density, based on observations from local monitoring sites within Shanghai. Rather than developing a universally generalizable forecasting model across the city, we focus on revealing how multiple urban and climatic factors jointly shape local mosquito abundance. In this light, we employ XGBoost as a flexible, data-driven approach to capture complex nonlinear relationships and pair it with SHAP to provide transparent, model-based explanations of variable importance. This framework emphasizes interpretive analysis, aiming to uncover how environmental and socio-demographic factors shape local mosquito abundance rather than serving as a large-scale predictive or causal model. Such insights can guide practitioners in allocating surveillance and control resources—for instance, by emphasizing source reduction in habitats conducive to outbreaks following particular weather patterns. Overall, the study offers an interpretable, fine-scale analytical framework that enhances understanding of urban mosquito dynamics and supports evidence-informed control strategies in rapidly urbanizing environments.

## Methods

### Study area

**Fig. 1:**
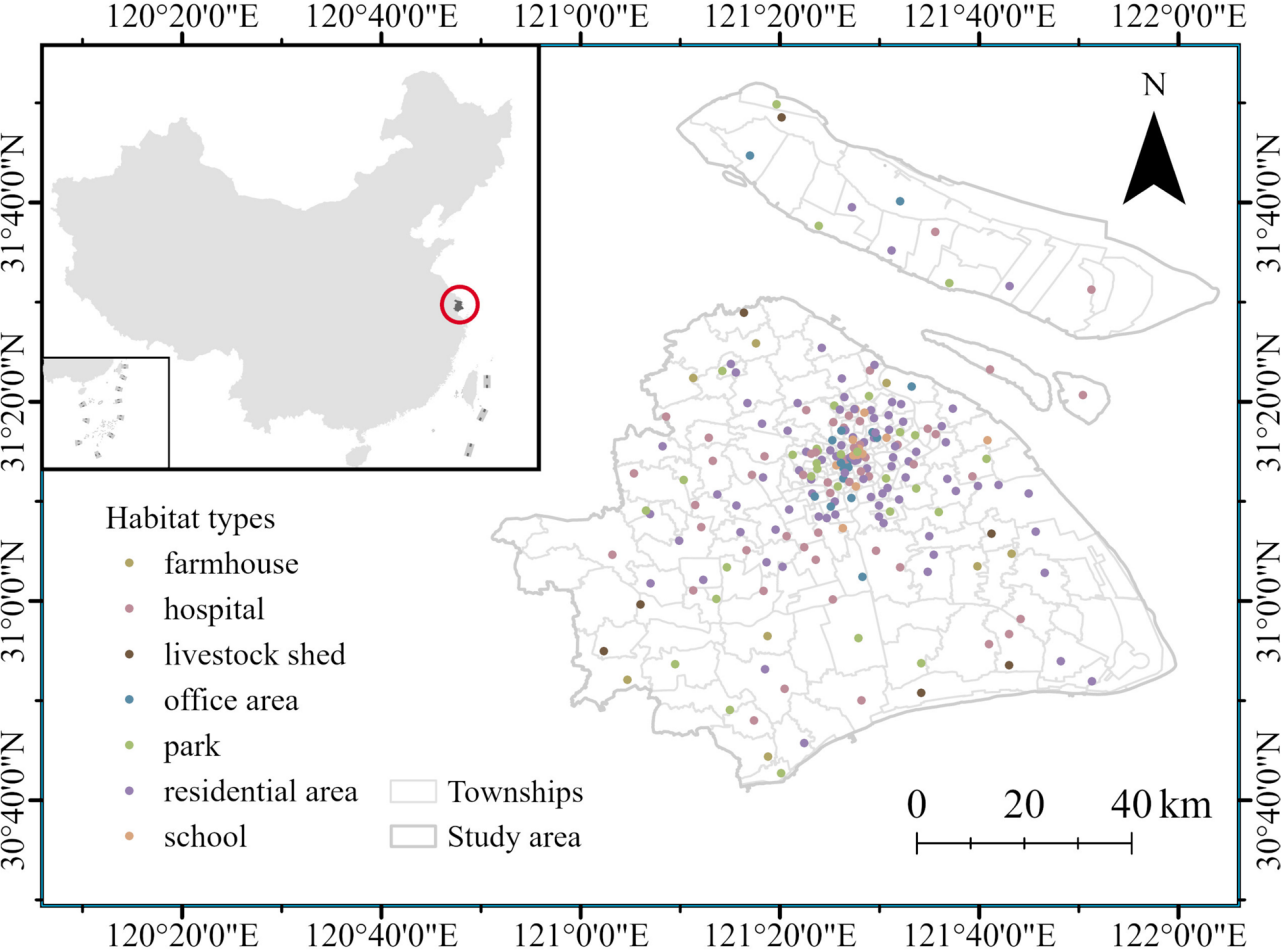
Research area

Shanghai, located on the eastern coast of China, has a subtropical monsoon climate, characterized by an average annual temperature of approximately 17 °C and annual precipitation > 1000 mm, with rainfall primarily concentrated from June to September [59]. The high degree of urbanization in Shanghai facilitates the local spread of mosquitoes [60, 61]. The study area encompasses all 16 administrative districts of Shanghai, with 239 mosquito-density monitoring sites across the city. Most monitoring sites were located in the urban core, while observations were much more sparsely distributed across suburban areas. These sites cover seven distinct habitat types, including parks, farmhouses, office areas, hospitals, schools, residential areas and livestock sheds, ensuring comprehensive representation of mosquito habitats in the urban environment (Fig. 1).

### Data description

#### Mosquito density data collection

Mosquito density data were obtained from 239 monitoring sites across Shanghai, with sampling conducted at approximately 10-day intervals from April to November 2023 (a total of 24 sampling cycles). We used a portable CO_2_-baited suction/light trap (model LTS-M0102, Professional version) deployed at fixed locations for 6 h per sampling. The device combines CO_2_, ultraviolet light and a human-mimicking lure to attract host-seeking adult mosquitoes, which are then drawn into a collection net by a fan. Compared with gravid traps that target ovipositing females, CO_2_-baited suction traps are designed to sample host-seeking adults, which is appropriate for relating abundance to human-biting risk in urban settings. Prior evaluations indicate that adding CO_2_ to light/suction traps, such as CDC light traps, can substantially increase adult catches, supporting our choice of a CO_2_-baited device for surveillance in this study [62–65]. After data validation and cleaning, a total of 5232 valid records from 218 sites were retained for analysis.

**Fig. 2:**
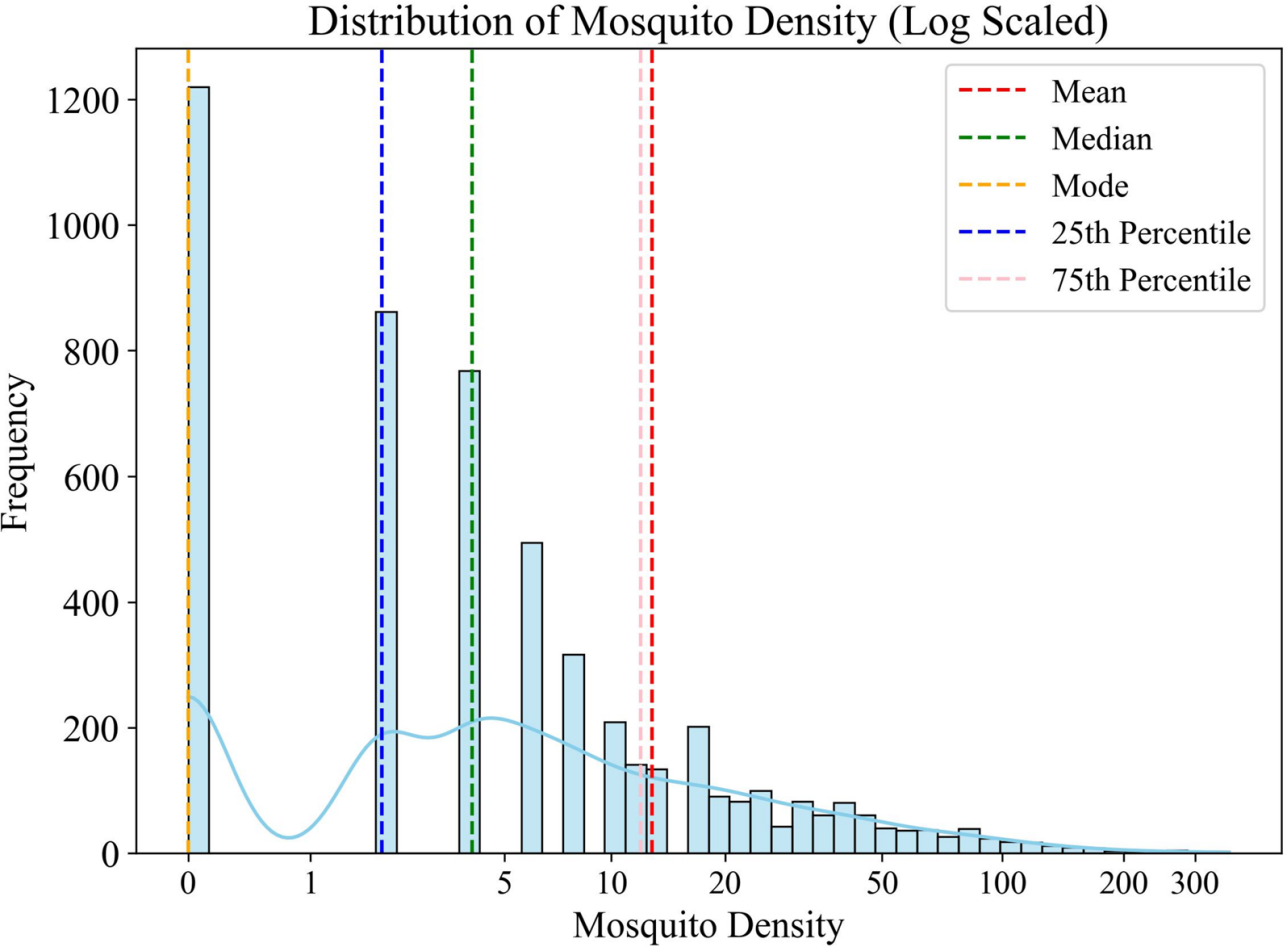
Statistical characteristics of mosquito density monitoring data

A preliminary statistical analysis (Fig. 2) revealed a right-skewed distribution of mosquito density. The mean mosquito density was 12.17, while the median and mode were 4.0 and 0, respectively, indicating that most sites had relatively low mosquito density. Further analysis showed that 25% of the monitoring sites had densities < 2.0, while 75% had densities < 12.0. The maximum density recorded was 364, and the standard deviation was 24.65, indicating high dispersion in the dataset. Given the complexity of urban environments, such variability is expected, reinforcing the need for advanced machine learning techniques such as XGBoost to model mosquito density, particularly in high-density areas.

### Meteorological data

Meteorological data were retrieved from the National Meteorological Station Database, which provides hourly observations from 11 meteorological stations across Shanghai. After data cleaning, the daily average temperature and cumulative precipitation were computed.

To spatially interpolate meteorological variables at mosquito monitoring sites, this study employed inverse distance weighting (IDW) for precipitation and Empirical Bayesian Kriging (EBK) [66] for temperature, with Digital Elevation Model (DEM) data obtained from the GEBCO organization [67].

### Environmental data

The study also incorporated multiple environmental factors, including mosquito habitat types, vegetation coverage, building height and demographic indicators.

Mosquito habitat types were categorized into seven distinct classes: park, farmhouse, office area, hospital, school, residential area and livestock shed.

NDVI was derived from Landsat satellite imagery [68] and resampled to 50-m resolution. Building height data were sourced from the 3D-GloBFP dataset [69]. The average building height within a 50-m radius of each monitoring point was calculated as an environmental feature. The flight range of *Ae. albopictus* is approximately 50–325 m [70–73], and the scales for NDVI and surrounding building height are based on this range.

Demographic data, including population density, compulsory education completion rate, proportion of older adults (aged ≥ 65 years) and proportion of children (aged ≤ 9 years), were extracted from the Seventh National Population Census at the community scale.

### Analytical model

#### Model construction and evaluation

To predict mosquito density, this study employs the XGBoost model, a widely used ensemble learning algorithm that iteratively constructs decision trees, refining each tree based on the residual errors of its predecessors. XGBoost optimizes performance by minimizing a loss function via second-order Taylor expansion by implementing pruning techniques to prevent overfitting [53]. XGBoost exhibits superior capabilities in capturing nonlinear relationships, performing automatic feature selection and enhancing computational efficiency, making it particularly suited for complex mosquito density prediction tasks. To evaluate the effectiveness of XGBoost, a comparative experiment was conducted using RF as a baseline. Both models were trained and tested using the same feature set and hyperparameter tuning strategy to ensure a fair comparison. Given the right-skewed count outcome, we additionally trained an XGBoost model with a Poisson count objective, which models the mean count per trap with a log-link and ensures positive predictions ($\widehat{{\mu }_{i}}$> 0). Throughout the study, we therefore compared three models: RF, XGBoost with the default squared-error objective and XGBoost with a Poisson count objective.

The explanatory variables incorporated in this study included meteorological factors (temperature, precipitation), environmental factors (NDVI, mosquito habitat type, building height) and demographic factors (population density, compulsory education completion rate, proportions of children and older adults). Habitat type was treated as a categorical variable via one-hot encoding (seven binary indicators) to avoid imposing ordinal structure. Given that the egg-to-adult development cycle of *Ae. albopictus* is approximately 2 weeks under warm conditions [74, 75], and that rainfall contributes to the formation and persistence of water-filled containers [29], we generated lagged temperature and precipitation variables at 0-, 3-, 7- and 14-day intervals (T, T_3d, T_7d, T_14d, P, P_3d, P_7d, P_14d) for sensitivity analysis.

Table 1 presents the descriptive statistics and spatial scale for the feature variables used in this study. To compare different lag windows, we performed sensitivity analysis to evaluate specifications using a single window (Only_0d, Only_3d, Only_7d, Only_14d), a specification excluding the 14-day terms (No_14d) and a specification including all windows (All) under the same training/validation protocol.

**Table 1:** Descriptive statistics of feature variables

Variables	Description	Max	Min	Mean	SD	Spatial scale
Habitat	Habitat types	NA (7 levels; one-hot encoded)				Site point
NDVI	Normalized difference vegetation index	0.41	− 0.02	0.13	0.07	50-m raster
Building_height	Average building height within 50 m (m)	80.34	0.00	21.68	9.29	50-m radius buffer
Pop_density	Population density (households)	9333	346	2647.17	1590.19	Community
CE_completion	Compulsory education completion rate	0.93	0.37	0.83	0.09	Community
Older	Proportion of older adults (aged ≥ 65 years)	0.42	0.02	0.16	0.07	Community
Children	Proportion of children (aged ≤ 9 years)	0.15	0.01	0.07	0.02	Community
P	Precipitation today (mm)	145.20	0.00	4.36	11.69	Site point
P_3d	Mean precipitation on the previous 3 days (mm)	67.97	0.00	5.45	9.22	Site point
P_7d	Mean precipitation on the previous 7 days (mm)	38.50	0.00	5.21	6.07	Site point
P_14d	Mean precipitation on the previous 14 days (mm)	22.26	0.00	4.91	4.77	Site point
T	Mean temperature today (°C)	33.00	7.56	23.05	6.17	Site point
T_3d	Mean temperature on the previous 3 days (°C)	32.96	8.67	23.10	5.82	Site point
T_7d	Mean temperature on the previous 7 days (°C)	31.22	9.64	23.18	5.40	Site point
T_14d	Mean temperature on the previous 14 days (°C)	30.64	9.12	23.29	5.18	Site point

The dataset was split into a training set (90%) and a test set (10%). All cross-validation and hyperparameter tuning were performed on the 90% training set; the 10% hold-out test set was kept untouched and used only once for final evaluation. Hyperparameters were tuned via grid search only during the training portion of each fold. The learning rate was searched over 0.01–0.30 (step 0.005) and the number of trees over 50–5000 (step 50). For RF, we tuned the number of trees under the same cross-validation protocol while keeping other parameters at their defaults. Since both XGBoost and RF determine split points based on feature ranking rather than numerical values, feature standardization was deemed unnecessary, simplifying preprocessing while maintaining model efficiency and accuracy.

Model performance was evaluated using four key metrics: root mean squared error (RMSE), mean absolute error (MAE), the coefficient of determination (R^2^), and Poisson deviance (PD), defined as follows:

$$RMSE=\sqrt{\frac{1}{n}\sum_{i=1}^{n}{\left({y}_{i}-{\widehat{y}}_{i}\right)}^{2}}$$

$$MAE=\frac{1}{n}\sum_{i=1}^{n}\left|{y}_{i}-{\widehat{y}}_{i}\right|$$

$${R}^{2}=1-\frac{\sum_{i=1}^{n}{\left({y}_{i}-{\widehat{y}}_{i}\right)}^{2}}{\sum_{i=1}^{n}{\left({y}_{i}-\overline{y }\right)}^{2}}$$

$$PD=2\sum_{i=1}^{n}\left[{y}_{i}\mathrm{ln}\left(\frac{{y}_{i}}{\widehat{{\mu }_{i}}}\right)-\left({y}_{i}-\widehat{{\mu }_{i}}\right)\right]$$

where $n$ is the number of samples, ${y}_{i}$ is the observed value of the sample, ${\widehat{y}}_{i}$ is the predicted value of the sample, $\overline{y }$ is the mean of the observed values, and $\widehat{{\mu }_{i}}$ is the model-predicted mean count used in the Poisson deviance (PD) ($\widehat{{\mu }_{i}}>0$). By convention, ${y}_{i}\mathrm{ln}\left(\frac{{y}_{i}}{\widehat{{\mu }_{i}}}\right)$ is defined as 0 when ${y}_{i}=0$.

RMSE is used to evaluate the overall prediction accuracy of the model. MAE reflects the average level of prediction error. R^2^ is used to assess the model’s explanatory power for the target variable. The closer the R^2^ value is to 1, the better the model’s performance. Poisson deviance quantifies goodness of fit on the Poisson count scale and is based on the difference between the fitted and saturated model; lower values indicate better calibration and fit for right-skewed count data. For comparability across folds, we report the mean Poisson deviance.

To mitigate overfitting to temporal structure and to evaluate out-of-sample generalization at the monitoring cadence, we implemented a temporally blocked cross-validation scheme, leaving out one dekad (approximately 10 days) at a time. The spatial distribution of monitoring sites was highly uneven, with dense coverage in central districts and sparse representation in suburban and peri-urban areas. As such spatial imbalance would make spatially blocked cross-validation unrepresentative and potentially biased, we did not apply a spatial blocking scheme. Although the analysis is not intended for forecasting, cross-validation was employed to assess model stability and ensure that the reported associations are not artifacts of temporal dependence [76–78].

In addition to cross-validation, we performed an exploratory out-of-time hotspot validation using July 2024 data. The 2023-trained model was applied without refitting, and Recall@K (K∈{5, 10, 15, 20, 25}) was computed to measure how well predicted high-risk sites overlapped with observed top-K% hotspots.

### SHAP-based feature interpretation

To improve the interpretability of the model’s prediction results, this study employed the SHAP method for feature importance analysis. SHAP is based on cooperative game theory and quantifies the contribution of each feature to the model’s prediction by calculating its marginal contribution in different feature combinations [54, 56]. The SHAP value for each feature is computed based on the splitting structure of the XGBoost model, as defined below:

$${\phi }_{j}=\sum_{S\subseteq F\left\{j\right\}}\frac{\left|S\right|!\left(\left|F\right|-\left|S\right|-1\right)!}{\left|F\right|!}\left[f\left(S\cup \left\{j\right\}\right)-f\left(S\right)\right]$$

where ${\phi }_{j}$ represents the SHAP value for feature $j$, $S$ is a subset of features, $F$ is the complete set of features, and $f\left(S\right)$ is the model prediction using only the features in set$S$.

This study used SHAP’s TreeExplainer to compute the SHAP values for the model and performed feature importance analysis on the test set. SHAP’s Detailed Summary Plot was used to visualize feature importance and analyze how specific changes in feature values affect the prediction results [56, 79]. To explore the model's prediction mechanisms across different scenarios, this study selected two representative samples for local SHAP analysis: one with the highest predicted value and one with the lowest. The sample with the highest predicted value helped identify the driving factors in high-mosquito-density areas, whereas the sample with the lowest predicted value revealed the feature impacts in low-density regions. For these key samples, SHAP Waterfall Plots were created to visually display each feature’s contribution to individual prediction values, thereby further validating the model’s rationale and providing deeper insights into the ecological interpretation of mosquito density.

### Analytical workflow

**Fig. 3:**
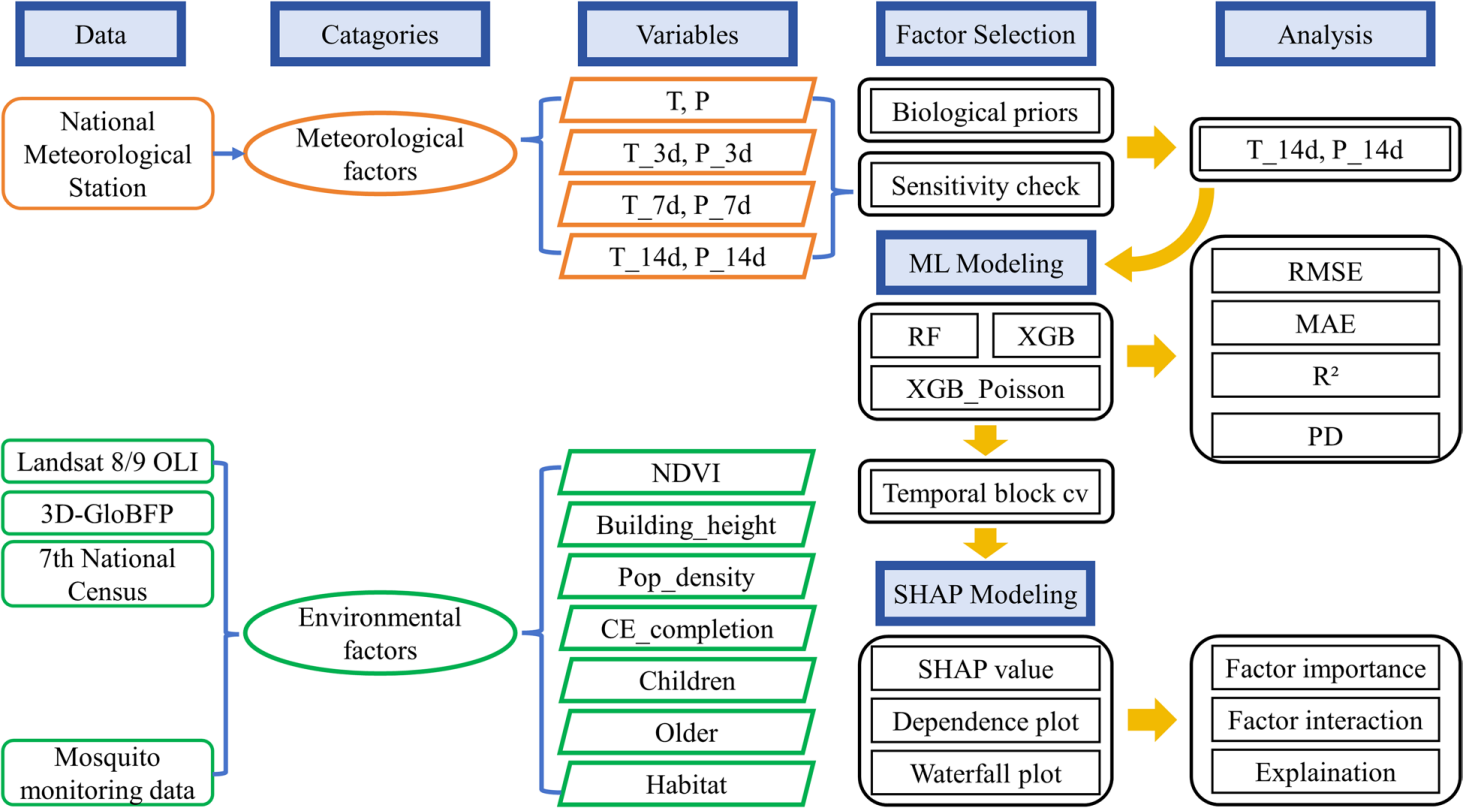
Overview of data sources, feature engineering, model training, evaluation and interpretability workflow

The overall methodology is summarized in Fig. 3, depicting the data sources, feature selection, machine learning models and interpretability analysis.

## Results

### Model performance evaluation

This study conducted sensitivity analysis on lag windows, and the results show the 14-day window consistently provided better out-of-sample accuracy and calibration (higher R^2^, lower Poisson deviance) than alternatives. Accordingly, the final model includes the 14-day mean temperature (T_14d) and the 14-day cumulative precipitation (P_14d) as meteorological predictors.

Regarding the test results on RF, XGBoost and XGBoost Poisson, the XGBoost Poisson model achieved the best overall accuracy and calibration (RMSE = 16.75, MAE = 7.10, R^2^ = 0.73, PD = 4.52) compared with XGBoost (RMSE = 18.96, MAE = 8.41, R^2^ = 0.66, PD = 9.31) and RF (RMSE = 23.36, MAE = 11.38, R^2^ = 0.48, PD = 8.23).

**Fig. 4:**
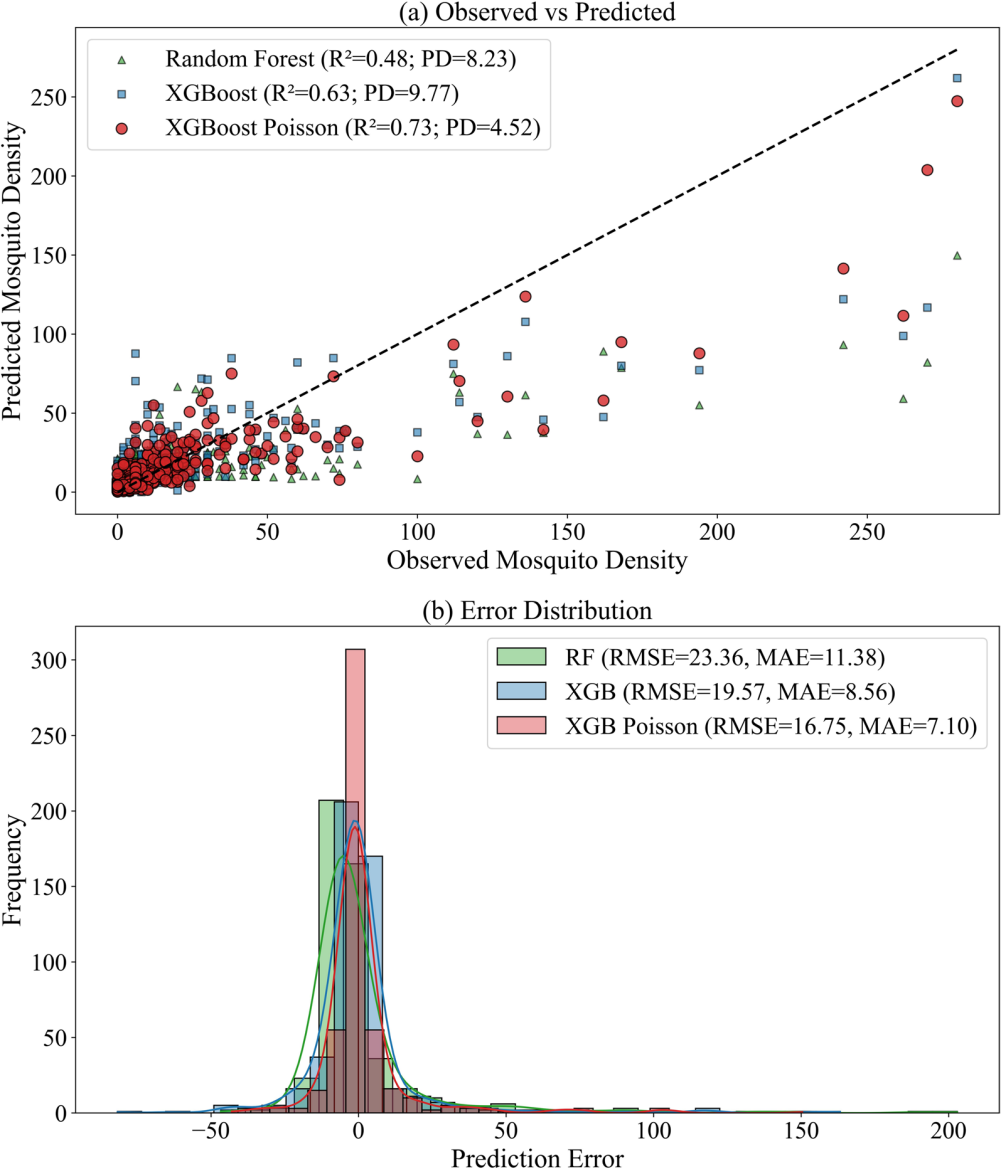
Comparison of predictions among RF, XGBoost and XGBoost Poisson. **a** Observed vs. predicted with the 1:1 reference line. **b** Error distributions (prediction errors) with overlaid density curves

Figure 4 visualizes the model behavior. In the scatter plot (Fig. 4a), RF and XGBoost tend to underpredict at the highest observed densities, whereas the XGBoost Poisson predictions lie closer to the 1:1 line and show both higher R^2^ and lower PD. The error histograms (Fig. 4b) further show a narrower residual distribution with lighter tails for the Poisson model, consistent with reduced extreme errors (including high-count underestimation) and improved overall calibration.

**Fig. 5:**
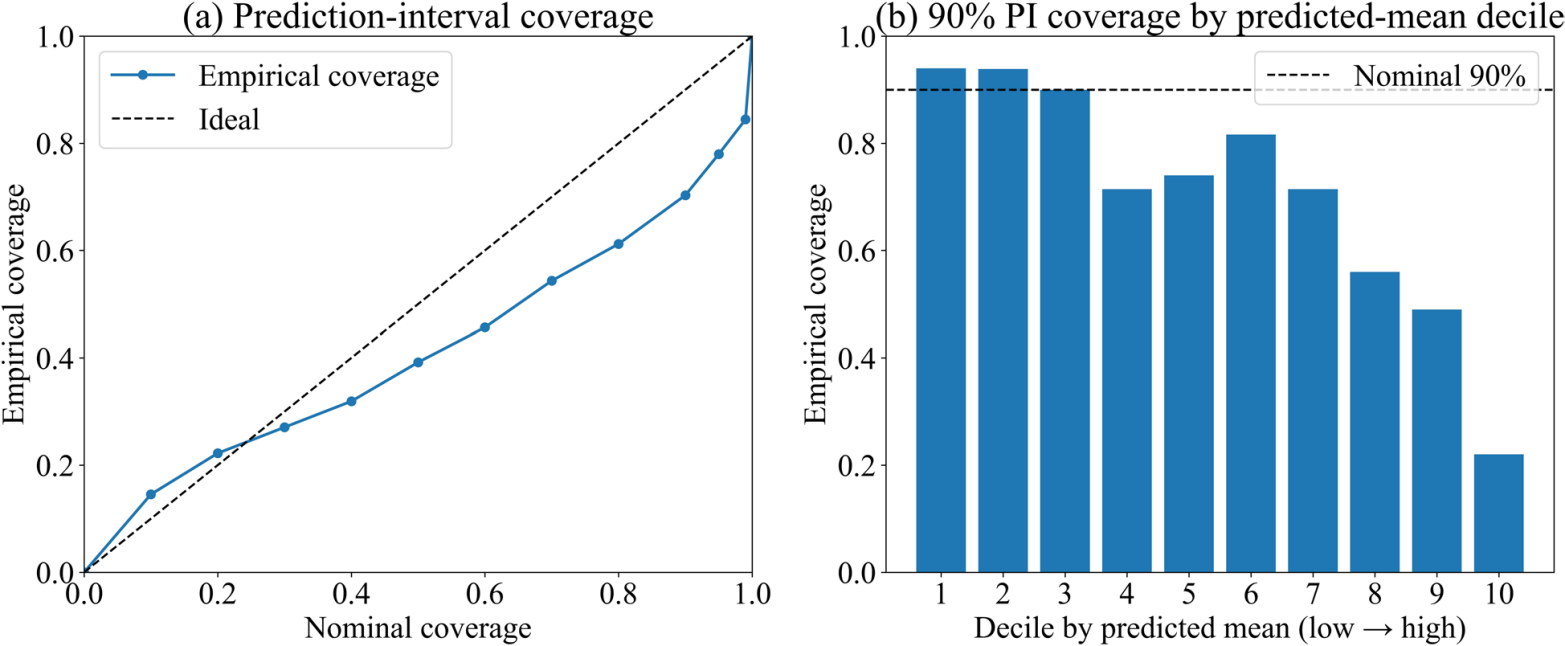
Prediction-interval coverage for the XGBoost Poisson model. **a** Empirical coverage versus nominal coverage for split-conformal prediction intervals. **b** Empirical coverage of 90% intervals by deciles of the predicted mean

We also evaluated empirical coverage of split-conformal prediction intervals to detect pockets of systematic under-/over-prediction. The empirical coverage curve falls below the ideal at higher nominal levels (Fig. 5a). For 90% intervals, overall coverage is below nominal; coverage by predicted-mean deciles is near nominal in the lower deciles but shows marked under-coverage in the upper tail (deciles 8–10) (Fig. 5b). These patterns indicate remaining difficulty at extreme counts and provide a basis for targeted refinements in high-risk, high-density settings.

To quantify uncertainty under temporal clustering, we report out-of-fold (OOF) performance with 95% confidence intervals (CIs) computed via dekad-blocked bootstrap under temporally blocked CV. Results are summarized in Table 2. Overall, the XGBoost Poisson model attains the lowest MAE and PD, with CIs indicating clear calibration gains over baseline XGBoost on the Poisson scale (non-overlapping PD intervals) and improved R^2^ relative to RF (non-overlapping intervals). Differences in RMSE remain modest with overlapping CIs across models, so we refrain from making stronger claims regarding RMSE.

**Table 2:** Performance with 95% CIs under temporally blocked CV using dekad-blocked bootstrap

Model	Metric	OOF point	Boot. mean	95% CI low	95% CI high
RF	RMSE	20.72	20.64	17.23	23.27
	MAE	10.90	10.89	9.68	12.01
	R^2^	0.33	0.32	0.27	0.36
	PD	8.97	8.95	6.01	11.86
XGBoost	RMSE	19.58	19.50	16.59	21.88
	MAE	9.37	9.35	8.10	10.57
	R^2^	0.39	0.39	0.33	0.44
	PD	14.73	14.73	11.39	17.87
XGBoost Poisson	RMSE	19.28	19.21	15.72	21.85
	MAE	8.15	8.15	6.99	9.19
	R^2^	0.42	0.41	0.37	0.46
	PD	8.06	8.04	6.06	9.85

Under temporally blocked CV, the XGBoost Poisson model retains R^2^ = 0.416 (out-of-fold; 95% CI 0.365–0.460), i.e. more than half of the hold-out R^2^ (0.73), suggesting reasonable temporal generalization at our monitoring cadence.

In the out-of-time hotspot validation (July 2024), the 2023-trained model recovered approximately half of the observed top-10% hotspots (Recall@10% ≈ 0.41–0.50), with recall increasing steadily to ~ 0.60–0.68 as the inspection range expanded to the top 25%. This indicates that the predicted ranking retained useful spatial localization of high-risk sites under unseen temporal conditions.

### SHAP analysis

To interpret the model’s predictions, this study employed SHAP to analyze the contribution of each feature to mosquito density predictions.

**Fig. 6:**
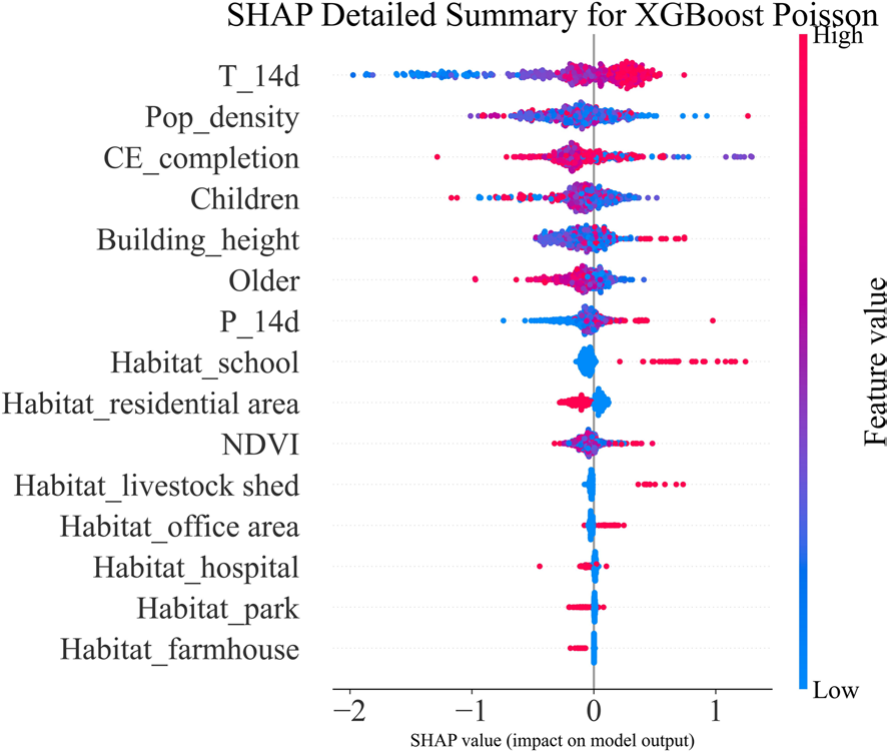
SHAP detailed summary plot. Features are ordered by global importance across samples

The SHAP detailed summary (Fig. 6) shows that the 14-day mean temperature (T_14d) has the largest overall impact on predictions, followed by population density (Pop_density) and compulsory education completion (CE_completion), with children’s proportion, building height and older-adult proportion forming the next tier. Precipitation during the previous 14 days (P_14d), NDVI and the one-hot-encoded habitat indicators exhibit smaller but non-negligible contributions. In terms of sign patterns visible in the plot, higher T_14d aligns with positive SHAP values, whereas higher CE_completion and a higher proportion of older adults align with negative SHAP values. Among habitat indicators, the residential area indicator shows a predominantly negative SHAP distribution relative to the reference; other categories are less prominent.

**Fig. 7:**
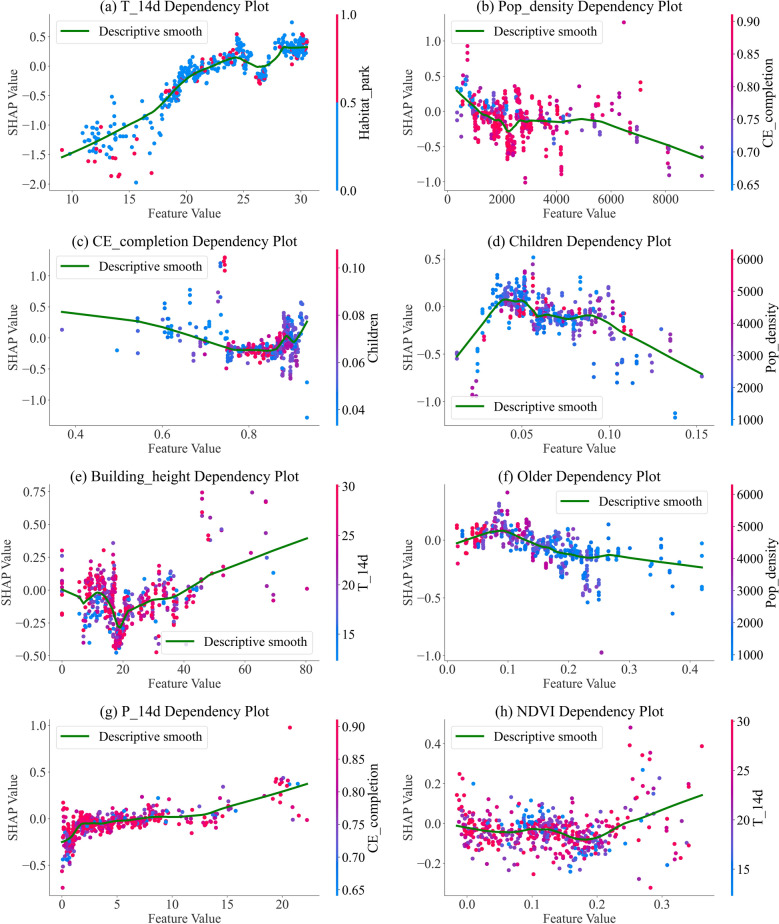
SHAP dependence plots for continuous predictors. **a** T_14d (mean temperature over the past 14 days), **b** Pop_density, **c** CE_completion, **d** proportion of children, **e** Building_height, **f** older proportion, **g** P_14d (mean precipitation over the past 14 days), **h** NDVI

Figure 7 presents marginal patterns for the continuous variables in the XGBoost Poisson model. The green curve is a descriptive smooth for visualization only; no formal inference is implied. Overall, the mean temperature over the past 14 days (T_14d) shows a positive, nonlinear trend with some flattening at the upper range (Fig. 7a). Higher population density was associated with slightly lower predicted mosquito density (Fig. 7b), and compulsory education completion (CE_completion) shows a negative gradient (Fig. 7c). The proportion of children shows an inverted-U pattern, with SHAP contributions increasing from low to moderate values and declining at higher values (Fig. 7d). Building height shows a shallow U shape with higher values tending toward positive SHAP contributions (Fig. 7e). The proportion of older adults decreases approximately monotonically (Fig. 7f). Precipitation over the previous 14 days (P_14d) shows a slight positive trend at larger values (Fig. 7g), and NDVI shows a mild positive gradient (Fig. 7h). For each panel, point colors indicate the most correlated auxiliary feature used for coloring and may suggest potential interactions; these are descriptive observations only.

**Fig. 8:**
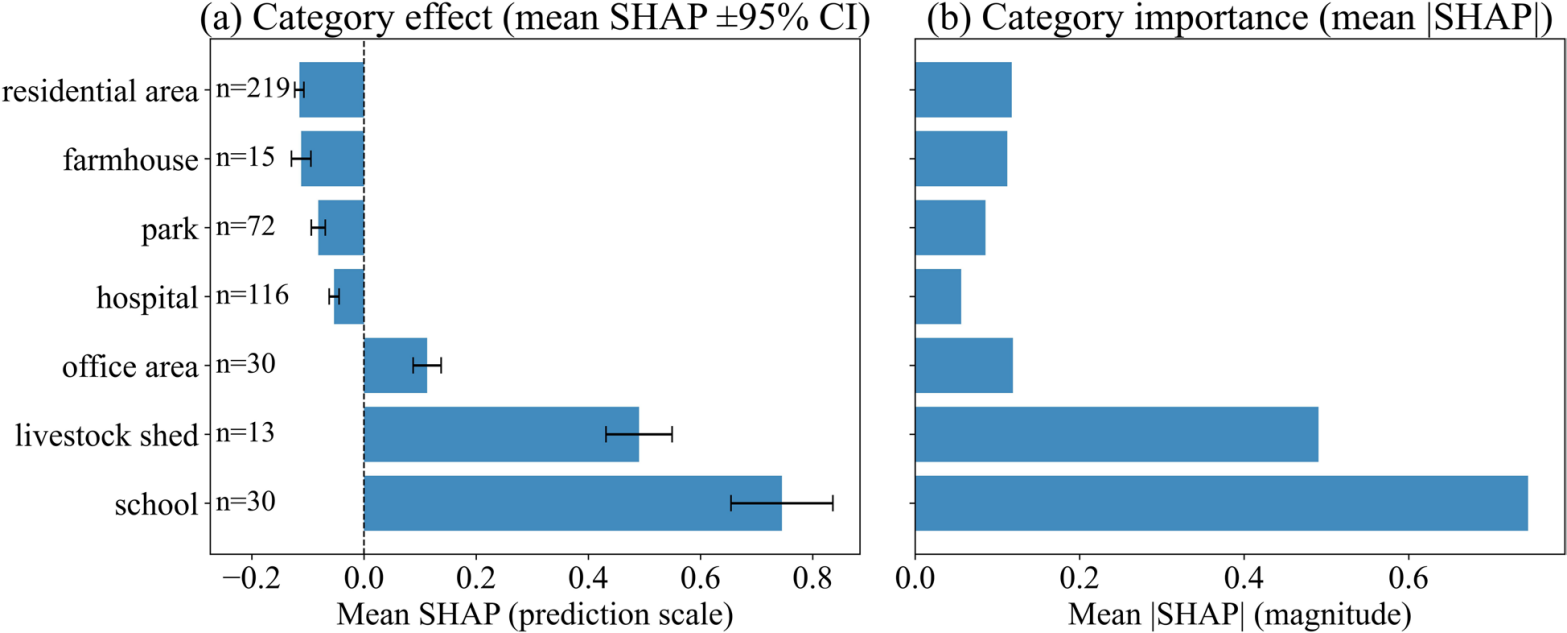
Category-level habitat effects (independent test set). **a** Mean SHAP value (prediction scale) by habitat category with 95% CIs. **b** Category importance measured by mean absolute SHAP

Figure 8 summarizes category-level SHAP contributions. In panel (a), the mean SHAP ± 95% CI shows schools (*n* = 30), livestock sheds (*n* = 13) and office areas (*n* = 30) shifting predictions upward (positive means), whereas residential areas (*n* = 219), farmhouses (*n* = 15), parks (*n* = 72) and hospitals (*n* = 116) shift predictions downward (negative means). To isolate contribution strength irrespective of sign, panel (b) reports mean |SHAP|: schools have the largest overall magnitude, followed by livestock sheds and office areas; among negative categories, residential areas show the largest magnitude. Sample sizes are shown to contextualize precision, and habitat was modeled as a categorical predictor via one-hot encoding.

**Fig. 9:**
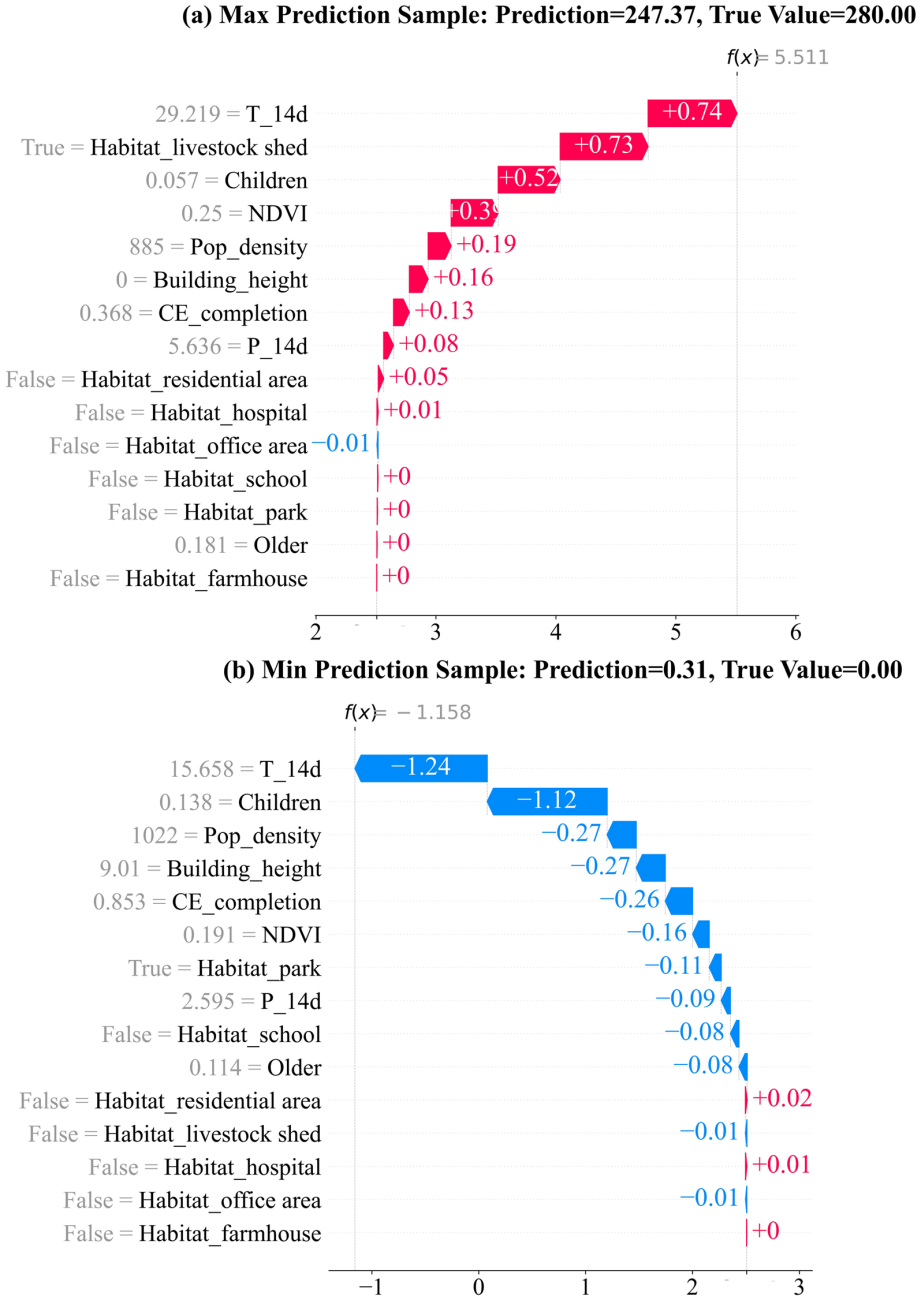
SHAP waterfall plots for two representative test-set samples. **a** Maximum prediction sample; **b** Minimum prediction sample

To visualize local contributions, Fig. 9 shows SHAP waterfall plots for two representative test-set samples under the XGBoost Poisson model. As the Poisson objective uses a log link, the bars in the waterfall plots are on the additive log-mean scale f(x), whereas the panel titles report back-transformed counts. For the highest-prediction sample (Fig. 9a; predicted 247.37, observed 280.00), the largest positive steps come from a higher 14-day mean temperature (T_14d), the presence of a livestock-shed habitat, an intermediate proportion of children and higher NDVI; smaller positive contributions are provided by lower population density, extremely low building height ($\approx$ 0 m) and lower compulsory education completion. For the lowest prediction sample (Fig. 9b; predicted 0.31, observed 0.00), negative steps are mainly driven by lower T_14d and a higher proportion of children, with additional negative contributions from higher education completion, park habitat, slightly higher population density, intermediate building height, lower NDVI and lower 14-day precipitation (P_14d). These two profiles are consistent with the global SHAP summary: temperature and habitat categories are primary drivers, with socio-demographic variables modulating predictions.

## Discussion

### Model performance and generalizability

The XGBoost–SHAP framework achieved reasonable within-sample fit and moderate temporal generalization (R^2^ ≈ 0.4) under time-blocked cross-validation. This reduction is expected, given the non-stationary nature of weather-mosquito relationships at short temporal scales. SHAP interpretation was derived from the independent test set to ensure stable and representative estimates of variable importance, while temporally blocked validation was used separately to evaluate model robustness across time. The spatial distribution of monitoring sites was highly uneven, with dense coverage in the urban core and sparse sampling in suburban areas. Because such imbalance would make spatially blocked validation unrepresentative and prone to bias, it was not performed.

To further assess operational relevance, an exploratory out-of-time hotspot validation was conducted using July 2024 data. The 2023-trained model recovered approximately half of the observed top-10% hotspots, and recall increased to approximately 0.65 when the inspection range expanded to the top 25%. Although predictive precision was modest, the results suggest that the model retained meaningful spatial localization of high-risk sites within the same monitoring network.

### Influence of key factors on mosquito density

Meteorological drivers remained foremost. The 14-day mean temperature (T_14d) showed the largest overall contribution and a positive, nonlinear pattern with some flattening at higher values, consistent with laboratory and field evidence that temperature accelerates development and biting up to warm optima before tapering [16–23]. Precipitation over the previous 14 days (P_14d) contributed positively at larger values, consistent with container creation and maintenance [17, 24–28], while remaining comparatively modest, plausibly reflecting urban drainage and source management in dense cities. The use of a 14-day window is consistent with mosquito biology; under warm conditions, egg-to-adult development typically spans approximately 2 weeks [74, 75].

Among environmental and demographic covariates, NDVI exhibited a modest positive effect, consistent with the hypothesis that vegetated resting sites facilitate adult survival [38–40]. A higher population density was associated with slightly lower mosquito density in this highly urbanized context, which may reflect better sanitation and more intensive vector management in the densest districts [80]. The proportion of older adults tended to be negatively associated, which might be related to lower daytime outdoor exposure [81]. The proportion of children showed an inverted-U nonlinearity. Although children tend to have longer daytime outdoor exposure and lower personal protection [82], the nonlinearity may be related to interactions with the built environment and population density (Fig. 8 c, d, color overlays) rather than a single monotone effect. Building height showed a shallow U shape, which may have been confounded by micro-climatic and vertical habitat effects (shade, wind sheltering, heat retention) that can either suppress or promote local abundance depending on the height range [83, 84].

Habitat categories, modeled as one-hot indicators, showed clear directional differences. Schools, livestock sheds and office areas shifted predictions upward on average, perhaps shaped by the day biting behavior of *Ae. albopictus *[85–87] and daytime aggregation of human or animal hosts. In terms of magnitude (mean absolute SHAP), schools ranked highest overall, probably because school hours result in sustained daytime aggregation of children.

Compulsory education completion (CE_completion) was negatively associated with predicted abundance. A possible reason could be that higher completion of education is potentially linked to greater awareness and practices that limit containers and exposure, such as improved waste handling, safer water storage and adoption of protective behaviors [82]. An alternative, and likely overlapping, explanation is that the variable may reflect broader socioeconomic and built environment conditions, including housing quality and municipal services, rather than education per se. Moreover, differences in education level, as an index of broader socioeconomic inequality, may coincide with areas of higher mosquito density and elevated disease risk, potentially reinforcing cycles of health and socioeconomic disparities.

### Operational insights for urban mosquito management

The findings offer indicative insights for vector-control prioritization in highly urbanized environments. In Shanghai, the model’s hotspot ranking suggests that focusing inspections on the top 10–25% of predicted high-risk sites may improve efficiency, while the 14-day meteorological lag provides a practical planning window for intensified surveillance and public messaging following rainfall or warm periods.

Habitat-level patterns highlight schools, livestock sheds and office areas as settings warranting particular attention, whereas residential and park sites may require continued but routine control. These interpretations should be regarded as data-driven signals rather than prescriptive rules, as operational strategies will need recalibration and validation under local ecological and programmatic contexts.

### Limitations and future work

While the framework provides meaningful interpretive insights into mosquito density variation, several limitations should be acknowledged. First, the model was trained and evaluated within a single city and season, which limits its capacity to capture broader spatiotemporal heterogeneity. Although temporal blocking was applied to reduce overfitting to short-term fluctuations, site-blocked cross-validation was not implemented because the monitoring network was spatially uneven, with dense coverage in central districts and sparse sampling in suburban areas. This imbalance would make spatial partitions unrepresentative and potentially biased. The exploratory out-of-time hotspot validation suggests that the model partially retains spatial localization of high-risk sites, yet predictive precision remains modest. Future work should include more spatially balanced sampling and independent testing in additional years, districts or cities to better evaluate transferability and avoid overfitting to local spatiotemporal patterns. Such efforts will help ensure that the framework’s insights remain robust and actionable under diverse environmental and urban conditions.

Additionally, trap- and measurement-related biases may have influenced the observed mosquito counts. The CO_2_-baited light suction traps used in this study primarily target host-seeking adults and may differentially capture *Aedes* relative to other genera, whereas they miss behaviors typically detected by gravid traps. Species composition, diel activity patterns and attractant type could therefore affect trap efficiency and abundance estimates. Moreover, several habitat categories were sparsely represented (e.g. livestock sheds, *n* = 13), thereby increasing uncertainty in category-level means. Expanding the monitoring network and achieving more balanced habitat sampling would enhance inference robustness and strengthen the framework’s operational relevance.

Several predictors in this study serve as proxies that may embed unmeasured confounding. For instance, the negative association observed with compulsory completion of education likely reflects broader socioeconomic and built environmental contexts, such as waste management, water storage practices and housing quality, rather than education itself. Future research could incorporate more detailed environmental datasets, including container surveys or infrastructure maintenance records, and, where causal inference is of interest, apply inferential frameworks with targeted controls.

Finally, the lag sensitivity analysis focused on 0-, 3-, 7- and 14-day windows, with the 14-day lag selected based on mosquito developmental biology and out-of-sample model performance. Rainfall intensity and cumulative indices beyond this window were not examined. Exploring event-based metrics, such as the frequency of heavy rain days, and multi-scale precipitation features may further clarify how rainfall dynamics influence mosquito breeding in dense urban settings with active drainage systems.

## Conclusions

This study developed a framework for estimating adult *Ae. albopictus* density in Shanghai by integrating XGBoost with SHAP-based model interpretation. SHAP results revealed that temperature was the dominant driver, followed by precipitation, vegetation and key urban covariates. Higher mosquito densities were associated with schools, livestock sheds and office areas, whereas residential and park sites tended to have lower values. Population and education variables reflected broader socioeconomic and environmental contexts associated with mosquito abundance. The model demonstrated moderate temporal generalization and localized high-risk hotspots with meaningful accuracy under out-of-time validation, suggesting potential utility for spatial prioritization in vector surveillance.

Several limitations should be acknowledged, including the use of data from a single city and season, uneven spatial coverage of monitoring sites and potential sampling biases associated with CO₂-baited traps. Future work should validate the framework across additional years and districts, incorporate richer environmental and infrastructural data and refine uncertainty estimates.

Despite these limitations, the study provides an interpretable, data-driven foundation for improving urban vector management and informing targeted control strategies in dense metropolitan settings.

## Data Availability

Data supporting the main conclusions of this study are included in the manuscript.
